# The MEC1 and MEC2 Lines Represent Two CLL Subclones in Different Stages of Progression towards Prolymphocytic Leukemia

**DOI:** 10.1371/journal.pone.0106008

**Published:** 2014-08-27

**Authors:** Eahsan Rasul, Daniel Salamon, Noemi Nagy, Benjamin Leveau, Ferenc Banati, Kalman Szenthe, Anita Koroknai, Janos Minarovits, George Klein, Eva Klein

**Affiliations:** 1 Department of Microbiology, Tumor and Cell Biology (MTC), Karolinska Instititet, Stockholm, Sweden; 2 RT-Europe Nonprofit Research Ltd, Mosonmagyaróvár, Hungary; 3 Microbiological Research Group, National Center for Epidemiology, Budapest, Hungary; 4 University of Szeged, Faculty of Dentistry, Department of Oral Biology and Experimental Dental Research, Szeged, Hungary; The University of North Carolina at Chapel Hill, United States of America

## Abstract

The EBV carrying lines MEC1 and MEC2 were established earlier from explants of blood derived cells of a chronic lymphocytic leukemia (CLL) patient at different stages of progression to prolymphocytoid transformation (PLL). This pair of lines is unique in several respects. Their common clonal origin was proven by the rearrangement of the immunoglobulin genes. The cells were driven to proliferation *in vitro* by the same indigenous EBV strain. They are phenotypically different and represent subsequent subclones emerging in the CLL population. Furthermore they reflect the clinical progression of the disease. We emphasize that the support for the expression of the EBV encoded growth program is an important differentiation marker of the CLL cells of origin that was shared by the two subclones. It can be surmised that proliferation of EBV carrying cells *in vitro*, but not *in vivo,* reflects the efficient surveillance that functions even in the severe leukemic condition. The MEC1 line arose before the aggressive clinical stage from an EBV carrying cell within the subclone that was in the early prolymphocytic transformation stage while the MEC2 line originated one year later, from the subsequent subclone with overt PLL characteristics. At this time the disease was disseminated and the blood lymphocyte count was considerably elevated. The EBV induced proliferation of the MEC cells belonging to the subclones with markers of PLL agrees with earlier reports in which cells of PLL disease were infected *in vitro* and immortalized to LCL. They prove also that the expression of EBV encoded set of proteins can be determined at the event of infection. This pair of lines is particularly important as they provide *in vitro* cells that represent the subclonal evolution of the CLL disease. Furthermore, the phenotype of the MEC1 cells shares several characteristics of ex vivo CLL cells.

## Introduction

Epstein-Barr virus can infect several human cell types. B lymphocytes are uniquely sensitive targets. Their differentiation marker CD21 serves as receptor for the virus. In the infected cells, interaction with cellular genes regulates the expression of viral genes. In a defined phase of differentiation a virally encoded growth program is expressed that induces proliferation. Practically all humans carry EBV. In health, the danger of proliferating EBV carrying B cells is constantly supervised and eliminated by immunological mechanisms [1].

Lymphoblastoid cell lines (LCLs) can be obtained by infecting B cells *in vitro*.[2] They can also emerge spontaneously from tissue explants that contain EBV genome carrying B lymphocytes when the *in vitro* condition modifies or eliminates the immunological cell mediated controls.[3] When the highly efficient control is compromised *in vivo* by immunosuppression, EBV positive B cell proliferations can occur such as in post transplant lymphoproliferative disease (PTLD) and AIDS associated lymphomas [4].

The viral growth program, latency Type III comprises nine EBV encoded proteins; EBNA1-6, LMP-1, -2A and -2B. Although their quantitative expression varies considerably, EBNA-2 and LMP-1 are essential for induction of proliferation. Presence of these two proteins is a marker for the proliferative EBV carrying B cell. Due to the requirement of specific transcription factors, the resident viral genes are expressed differently as the B cell proceeds in the differentiation path and it is also determined by the differentiation phase of B cell at the event of infection.[1], [5], [6], [7] When the virus infects B cells that are outside the appropriate differentiation window, either EBNA-2 or LMP-1, or both are not expressed. These “restricted expressions” are denoted as latency Type 0, I, IIa, IIb. The fate of these cells differs considerably. Only the Type IIa cells proliferate and develop malignancy; generated by a complex interaction with microenvironment as in EBV positive Hodgkin’s lymphoma, HL. In the autoregulatory circuit the cells with Type IIa latency elicit a granulomatous tissue reaction that produces growth factors [1], [8].

In CLL disease, B lymphocyte clones proliferate. These originate from self-renewing hematopoietic stem cells, stimulated by autoantigens and by the stroma cells.[9], [10] The clinical course of disease differs remarkably depending on the mutation status of immunoglobulin (IGHV) genes, expression of CD38 and zeta-chain-associated protein kinase 70 KDa and ZAP-70 [10].

Recently, attention was directed to the subclonal heterogeneity of the CLL populations with emerging dominant clones that lead to distinct periods in the progression of the disease.[11] In some patients progression to the aggressive prolymphocytic cell profile occurs in the terminal stage.[12] Rarely, progression is accompanied by phenotypical cellular changes resulting in HL, PLL or diffuse large B cell lymphoma, DLBCL-like diseases [13], [14], [15], [16].

EBV is not involved in the pathogenesis of CLL. The CLL cells can be infected *in vitro* but only rare clones are induced to proliferate. The infected cells express a viral program that lacks LMP-1, we referred to it as latency Type IIb.[1] In contrast, *in vitro* infected PLL cells could express the complete growth program [17].

Cells of occasional CLL patients were transformed to LCLs, when infected *in vitro*.[18] In addition, LCLs could be established from explanted CLL cells even without experimental infection.[19] The origin of the MEC1 and MEC2 lines was similar. They grew from subsequent explants of the patient.[20] As reported in the original and in several subsequent publications, the phenotype and biological behavior of the 2 cell lines differ.[21], [22], [23] We extended the study of this unique pair of lines.

Acquisition of EBV by CLL cells in different stages of the disease provided these *in vitro* lines with features that reflected the clinical status of the patient at the time of their origin. Two features can be singled out from our analyses that are in line with the development of the disseminated final stage. MEC2 but not the MEC1 cells express CD38 that is a marker for progression in CLL and MEC1 express CXCR4 that is present on CLL cells, while it is conspicuously reduced in the MEC2 line. According to a recent report, the expression of the suppressor microRNAs, MiR-15/-16 differs in the two MEC lines. Their processing and maturation are impaired in the MEC2 cells.[21] This may provide an important property that contributes to the aggressive behavior of the cell of origin which with the contribution of EBV grew *in vitro* and established as the MEC2 line.

## Materials and Methods

### Cell culture

The two lines, MEC1 and MEC2 were established from the spontaneous outgrowth of explanted CLL cells on subsequent occasions with one year interval when the disease underwent marked prolymphocytoid transformation.[20] The characteristics of the ex vivo cells were described in the original publication. The disease was diagnosed as CLL though the cells were not typical in that they had strong surface immunoglobulin expression and lacked CD23. LCL derived from cord blood, CBM1-Ral-STO, and the Burkitt’s lymphoma (BL) line, Daudi, were used as EBV positive and the Ramos line as EBV negative control.[24], [25], [26] The cells were cultured in RPMI 1640 supplemented with 10% heat-inactivated FBS, 100 units/ml penicillin, and 100 µg/ml streptomycin in humidified incubator at 37°C and 5% CO_2_.

### Immunoglobulin gene analysis

PCR amplification of IGH gene rearrangements was performed on genomic DNA using subgroup-specific framework 1 (FR1) primers, together with a consensus IGHJ primer as previously described.[27] Sequences were analyzed using the IMGT database and the IMGT/V-QUEST tool (http://www.imgt.org) [28], [29].

### Immunofluorescence staining

The details of the staining and imaging were described previously.[30] For single staining, mouse monoclonal antibody (mAb) specific for EBNA-2 (PE-2, culture supernatant prepared in our laboratory), for LMP-1, CS1-4, mixture of 4 mAb (Novocastra Laboratories Ltd, UK) or mAB S-12 (prepared in our laboratory) and for simultaneous detection, isotype specific anti LMP-1, S-12 (IgG_2a_) and anti EBNA-2, PE-2 (IgG_1_) were used. Alexa fluor 488 and 594 labeled isotype specific goat anti mouse IgG_1_ and IgG_2a_, accordingly (Life technologies, USA) were used as secondary antibodies.

### Immunoblotting

The cells were lysed in sodium dodecyl sulfate (SDS) gel-loading buffer. Lysates corresponding to 1.5×10^5^ cells were loaded from CBM1-Ral-STO and Ramos. 5×10^5^ cells were loaded from MEC1 and MEC2. Immunoblotting was performed with the antibodies, PE-2 (EBNA-2), CS1-4 (LMP-1), and 3H2- E8 (Blimp-1, Novus Biologicals), as described previously.[31] As a control for protein loading, mAb specific for β-actin, clone AC-15 (Sigma–Aldrich, USA) was used.

### Real Time Quantitative PCR

The primer sequences and PCR conditions used were described in our earlier publication and also shown in Table S1.[31] GAPDH served as endogenous control.

### Control DNA sequencing

Genomic DNAs were amplified with PCR using the primers and PCR conditions listed in Table S1. Both strands of the PCR products were sequenced on a MegaBACE DNA sequencing system (GE healthcare) using dye-labeled ddNTPs, according to the manufacturer’s instructions.

### Automated genomic sequencing of sodium bisulfite-treated DNA

We used the method as described earlier.[32] Primers used for the amplification of Cp are shown in Table S1.

### Terminal repeat fragment analysis

Genomic DNAs were digested with BamHI and the resulting fragments were separated on a 0.8% agarose gel, blotted to a Hybond N membrane and hybridized with a DIG-dUTP-labelled PCR product generated from the B95-8 prototype EBV genome with primers 5′-GTA TGC CTG CCT GTA ATT GTT G-3′ and 5′-ACG AAA GCC AGT AGC AGC AG-3′.

### Flow Cytometry

The cells were washed in cold PBS containing 2% FCS and then stained with FITC-, or PE-, or PE-Cy5-conjugated mouse anti-human monoclonal antibodies. The following specificities were used: CD5, CD10, CD11c, CD19, CD20, CD21, CD23, CD25, CD27, CD38, CD45, CD54, IgM, HLA-ABC, HLA-DR and CD19 (Becton Dickinson, Ca). Antibodies detecting CXCR4, CXCR5, CCR7 and CCR10 (R & D Systems, MN) were also used. Ten thousand events were collected on a FACScan flow cytometer, and the results were analyzed using CELLQUEST (Becton Dickinson) software.

### Exposure to IL-21 and to CD40L

IL-21: As described in our earlier publication, IL-21 (100 ng/ml, PeproTech EC, UK) was added to cultures containing 0.16×10^6^ cells/ml.[31] The cultures were readjusted on third day to 0.16×10^6^ cells/ml and IL-21 was re-added. The cells were harvested on the 6^th^ day for analysis.

CD40 ligand, CD40L: 0.5×10^6^ irradiated (15,000RAD) L or CD40L-L cells were plated in wells of a 24 well plate and used 24 hours later. Equal number of MEC1 and MEC2 cells were seeded on the monolayer and incubated for 3 days at 37°C and 5% CO_2_ [33].

## Results

### Identity of the lines

We list the characteristics of the MEC1 and MEC2 cells used in the current study. The analysis includes features that correspond well with those described in the original publication [20].

The derivation of the cell lines from the patient’s CLL cells was proven by the identity of the DNA rearrangement in the IgH loci in the ex vivo sample and in the lines. Both lines belonged to the VH4 family. The cell lines used in the present study carry IGHV4-59/IGHD2-21/IGHJ6 gene rearrangements with 94% identity to germline as it is described in the original publication [20].

The MEC2 cells are larger than the MEC1 cells. MEC1 cells are mainly solitary. They form few and small aggregates. The social behavior of MEC2 cells is different. The majority of the cells create large aggregates. Morphological and proliferation properties and the surface marker profiles of the cells corresponded at large to that reported originally [20].

### The lines carry the same EBV strain but the infection events differed

Based on their sequence of the Cp region from 10480 to 11461 (European Nucleotide Archive accession numbers for MEC1 and MEC2 are HG380070 and HG380071 respectively), the two lines contained the same EBV strain, differing from the widely used prototype, B95-8 [34].

The cells carry predominantly latent episomal EBV genomes. The terminal repeat analysis showed single fragment with different size in the lines (Figure S1). Therefore we can conclude that the cells of origin were infected at different occasions.[35] This was in accordance with the difference in the promoter usage for EBNA-2 expression.

### Expression and regulation of the EBV encoded latent proteins, EBNA-2 and LMP-1

**Figure 1:**
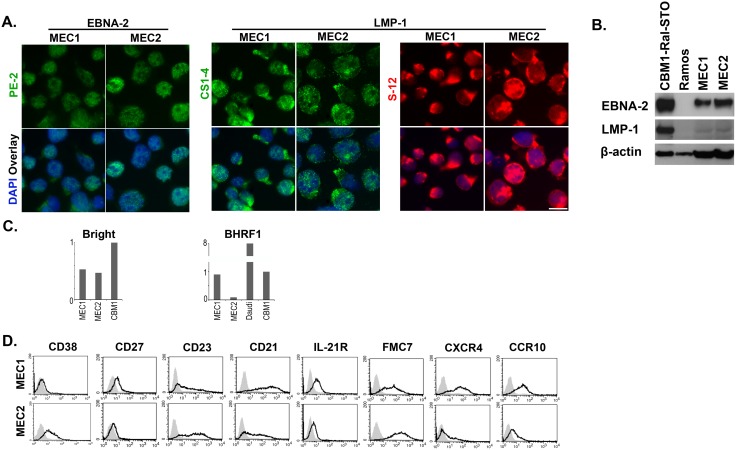
Comparison of the MEC1 and MEC2 cells. (A) Expression of EBV encoded proteins EBNA-2 and LMP-1 by immunofluorescence; magnification (×100), scale bar 25 µm. Note: the MEC2 cells are larger. (B) Expression of EBNA-2 and LMP-1 by immunoblotting; positive control: CBM1-Ral-STO, negative control: Ramos. 1.5×10^5^ cells were loaded in control lanes and 5×10^5^ were loaded in MEC1 and MEC2 lanes. Note MEC2 expresses higher amount of EBNA-2. (C) Expression of Bright and BARF1 by Q-PCR. (D) FACS analysis of surface markers that are differently expressed in the 2 lines.

All cells in the MEC1 and MEC2 lines express both EBNA-2 and LMP-1. Thus they correspond to Type III latency (Fig. 1A) (also see Figure S2). Two antibodies were used for LMP-1 detection, CS1-4 and S-12 and they localized mainly to the cell membrane. The pattern with CS1-4 was dotted while it was patchy with the S-12. The LMP-1 staining showed also that MEC2 cells are larger and analysis of the populations shows a shift to the larger sizes in the MEC2 culture (Figure S2). The immunoblots detected higher level of EBNA-2 in the MEC2 cells (Fig. 1B).

**Figure 2:**
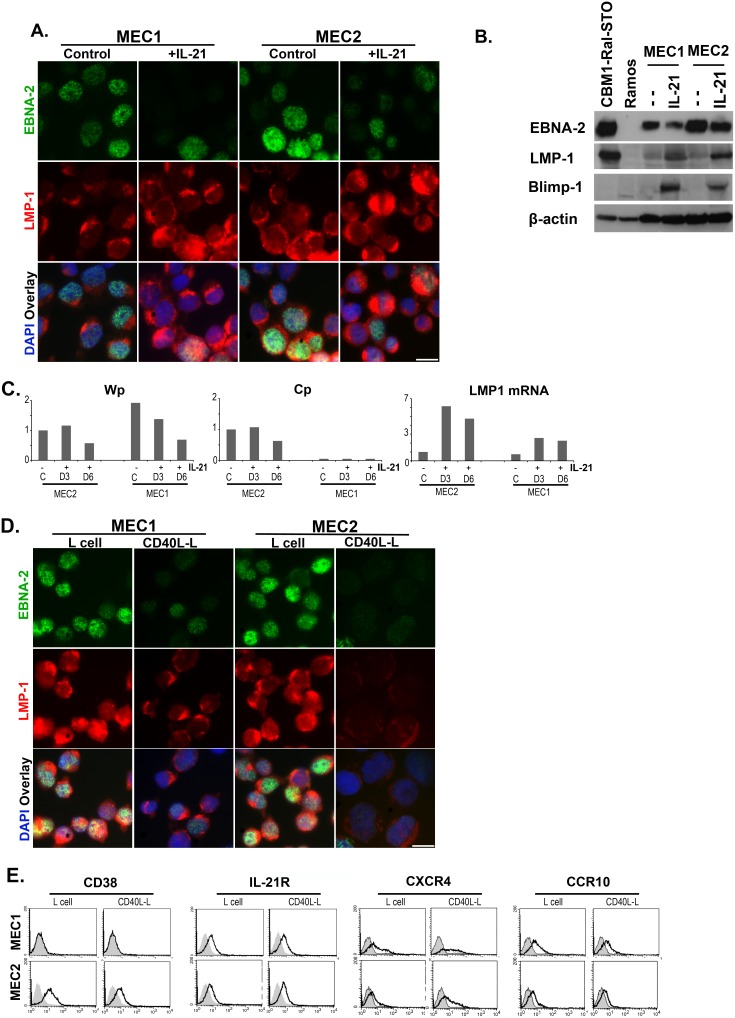
The effect of IL-21 and CD40L exposure on MEC1 and MEC2 cells. Expression of EBNA-2 and LMP-1 in IL-21 treated cells (A, B). (A) Simultaneous immunofluorescence staining of EBNA-2 (Green) and LMP-1 (Red); magnification (×100), scale bar 25 µm. Note the downregulation of EBNA-2 and upregultion of LMP-1 after IL-21 treatment. (B) Expression of EBNA-2, LMP-1 and Blimp-1 by immunoblotting; positive control: CBM1-Ral-STO, negative control: Ramos. 1.5×10^5^ cells were loaded in the control lanes and 5×10^5^ were loaded in both untreated and IL-21 treated MEC1 and MEC2 lanes. Note low expression of EBNA-2 and high expression of LMP-1 after IL-21 treatment and induction of Blimp-1 after IL-21 treatment. (C) Activity of the W and C promoters that regulate EBNA-2 expression and LMP-1 mRNA expression by Q-PCR. Note the difference in EBNA-2 regulation; the MEC2 cell uses both Wp and Cp while in MEC1 only Wp is active. (D) Expression of EBNA-2 and LMP-1 in cells exposed to CD40L. Simultaneous immunofluorescence staining; for details see (A). Note: EBNA-2 and LMP-1 are downregulated by CD40L in both lines. (E) CD40L induced modulation of surface marker by FACS analysis.

Analysis of the promoter activities confirmed the Type III latency with difference in the transcription program of EBNA-s (Fig. 2C). In MEC1 cells only the W promoter, Wp, while in the MEC2 cells Cp and Wp were active. Wp activity was twofold higher in MEC1 than in MEC2. Dual usage of Wp and Cp is regular in the Type III LCLs.[36] The LMP-1 mRNA was expressed in both lines but it was lower in the MEC1 cells. Q promoter, Qp was silent in both lines.

The difference in the lines with regard of EBNA-2 regulation does not seem to be determined by their methylation pattern since the genomic sequence of Cp region was unmethylated in both (Figure S3).

Similarly, it is unlikely that the B cell specific transcription factor ARID3A/Bright that is known to upregulate Cp activity accounts for this difference because the mRNA levels were similar in MEC1 and MEC2. It was about half in comparison to a regular LCL, CBM1-Ral-STO (Fig. 1C) [37].

Kelly et al. suggested that BL cells which use exclusively the Wp are particularly resistant to apoptosis because of Wp driven expression of the viral bcl2 homologue BHRF1.[38] The MEC lines do not show this correlation. Both MEC lines express low level of BHRF1, though in MEC1 the Wp activity is twofold higher (Fig. 1C). In MEC1 the BHRF1 level was similar to the Cp user LCL, CBM1-Ral-STO and it was even lower in the MEC2 cells that use both Wp and Cp.

### Phenotypic differences determined by surface marker expression

Expression of surface markers by the MEC lines reported in the original publication as well as additional markers is summarized in Table S2. Selected FACS profiles are shown in Fig, 1D. The B cell markers, CD19, CD20 and HLA-ABC, HLA-DR, CD30, CD54/ICAM-1 and CCR7 were detected with similar profiles on both lines, while they differed in the expression of CD38, CD27, CD23, CD21, IL-21R, FMC7, CXCR4, and CXCR10. We discuss here the markers that may be relevant to the biological behavior of the cells.

CD38 is a marker for poor prognosis as it indicates activation and recent proliferative history of the CLL cells. CD38 positive cells in the blood are assumed to be recent emigrants from the proliferation centers; lymph nodes and bone marrow.[39] CD38 was expressed by the majority (64%) of MEC2 while it was absent on the MEC1 cells. The difference indicates that the lines arose from different subclones and it is in accordance with the clinical status of the patient; the disease being more extended at the time of the derivation of the MEC2 line.

CD27, the memory B cell marker is expressed by CLL cells.[40], [41] It is present on a significant proportion of MEC1 cells (23%) but not on MEC2 cells (2%).

CD23 is a B cell activation marker. It is expressed by LCLs.[42] CLL cells also express CD23 and has positive correlation with CD38.[43], [44], [45] In accordance, it was detected on lower proportion on MEC1 cells (51%) than on MEC2 cells (83%).

CD21, the complement receptor, is expressed by CLL cells.[19] It serves as receptor for EBV. Its expression was higher on MEC1 (91%) than on MEC2 (56%) cells. This difference is in good correlation with its expression on CLL and PLL. It was reported to be lower on PLL than on CLL cells [46], [47].

IL-21R was shown to be inversely correlated with CD38 expression in CLL cells.[48] Similar tendency was observed on the MEC lines. The CD38 negative MEC1 line had higher (51%) expression than the CD38 positive MEC2 line (28%).

FMC7 is strongly expressed by CLL cells when they proceed to prolymphocytoid transformation.[44] Although a major proportion of (81%) the CLL cells in the ex vivo sample was FMC7 positive, the established MEC1 line contained only 8% positive cells.[20] In our present analysis, the majority of the MEC1 cells expressed FMC7 (74%) and all MEC2 cells expressed this marker. The patient’s CLL cell that generated the MEC1 line may have been in an early transition towards the PLL stage and progressed further *in vitro*.

Chemokine receptors and adhesion molecules guide the migration of CLL cells between the tissues and the circulation.[49], [50] Resting B cells in the blood have high expression of CXCR4 and CCR7 and low expression of CCR10. On EBV immortalized LCL cells, CXCR4 and CCR10 are expressed reciprocally, low CXCR4 and high CCR10 [51].

CXCR4 was shown to be present on resting CLL cells in the blood. The recently emigrated cells from the proliferation centers have low levels.[11], [52] The majority of MEC1 (84%) cells but only a small proportion of MEC2 (26%) cells express CXCR4. This can be related to the aggressive clinical stage of the disease, when high cell numbers are discharged from the proliferation centers. Expression of this marker is similar in the MEC2 and LCL cells.

CCR10 expression was higher on MEC1 (75%) than on MEC2 (26%) cells. Neither the CXCR4 nor the CCR10 markers conform with the phenotypic relationship with LCL cells and with EBV positive B cells localized at the periphery of tonsil in infectious mononucleosis [51].

CCR7 is similarly expressed by MEC cells and LCL cells.[51] The relationship between the MEC lines and LCLs with regard to the chemokine receptor doesn’t provide any clue to their biological behavior.

The surface marker profile of the MEC1 line has many similarities with CLL cells. At the time of its establishment, the patient’s clinical condition did not progress yet and as published earlier, similar to CLL cells, MEC1 could grow in immunosuppressed mice while the MEC2 cell did not.[22], [23] Some of the markers, such as the high Ig expression, the transformation to Type III cells by EBV infection, indicate that at the time of establishment of MEC1, the disease already entered progression to PLL. Subsequently, cells in further stage of transformation dominated and lead to the aggressive clinical stage.

### Influence of IL-21 and CD40 Ligand on the expression of EBV encoded proteins

Soluble factors produced by activated CD4^+^ T cell was shown to influence the expression of EBV encoded proteins and thus change the EBV latency type.[53] IL-21 is known to induce plasmacytoid differentiatioin of LCLs, and plasma cells do not support Type III expression.[31], [54] Treatment of LCL with IL-21 downregulated EBNA-2 expression thus it changed the latency from Type III to Type IIa. Concomitantly, the cells ceased to proliferate. IL-21 also upregulated LMP-1 protein expression. Similar changes were induced in the MEC lines (Fig. 2A, & B). The IL-21 induced plasmocytoid differentiation was substantiated by expression of Blimp-1 (Fig. 2B).

The changes were confirmed by the corresponding promoter activities (Fig. 2C). Wp activity decreased in MEC1 cells and both Wp and Cp activity decreased in the MEC2 cells and the LMP-1 mRNA level was elevated in both lines.

Similar to earlier report, co-culture of LCL with CD40 ligand (CD40L) expressing L cells reduced EBNA-2 and LMP-1 expression, in the MEC lines (Fig. 2D).[33] On the basis of the known effects of CD40L on the differentiation of B cells, both normal and LCLs, it is likely that alteration of the EBV encoded protein expression is a consequence of change of differentiation towards germinal center and memory B cells.[33], [55] Co-cultivation with L cells (without CD40L) elevated also the LMP-1 expression on the MEC cells, though to a lesser degree (Fig. 2D). For base line LMP-1 expression see Fig. 2A.

### CD40L induced modulation of CD38 and chemokine receptors, CXCR4 and CCR10

CD40L exposure resulted in upregulation of CXCR4 in the MEC lines (Fig. 2E). This upregulation of CXCR4 might be due to CD40L induced downregulation of EBNA-2 and LMP-1.[51] Similar to CLL cells, slight upregulation of IL-21R and downregulation of CD38 was noted in MEC2 cells in response to CD40L.[48] CD40L induced no significant change in IL-21R and CD38 expression in MEC1. However, CCR10 was downregulated following CD40L exposure in both lines. CD40L induced change of differentiation is reflected by the change of surface marker phenotypes in MEC cells.

## Discussion

EBV is not involved in the pathogenesis of CLL. Presently it is emphasized that subclonal variation and selection lead to the evolution of the disease with alteration of the biological behavior, activation state and proliferation of the cells.[9], [10], [11] In some cases EBV carrying subclones have been detected by their capacity to proliferate *in vitro*; giving rise to LCLs with proven CLL origin.[56], [57] *In vitro* infected CLL cells exhibit an unusual viral latency, Type IIb; the cells express EBNA-2 but not LMP-1 and they do not proliferate. EBV positive B cells with Type IIb program were detected in tissues of PTLD, IM and in EBV infected humanized mice.[1] We detected rare cells with Type IIb latency in *in vitro* infected cord blood derived lymphocyte population.[30] In contrast to the CLL cells, EBV can induce *in vitro* proliferation of PLL cells.[17] It is important to note that even when EBV positive subclones were detected in the CLL population, these cells did not lead to development of EBV positive disease.[19] This indicates that the proliferation of EBV carrying B cell can be efficiently controlled even in the severe leukemia condition. This is in contrast with the development of EBV positive B cell proliferation in PTLD, when the immune response is compromised due to the immunosuppressive treatment [4].

EBV carrying lines have been established from CLL cells in a few experiments.[20], [58] Similar to the MEC1 and MEC2, cell lines with somewhat differing properties were established earlier from explanted lymphocyte samples of a CLL patient.[19] During the 5 final years of the case history, lines were established from cultures to which the anti-viral agent phosphonoformate and virus-neutralizing antibodies were added. These prevented virus release and infection of B cells *in vitro*. One group of lines was the descendants of one clonal CLL cell that carried the virus *in vivo*. It was estimated that these cells represented 0.1% of the CLL cell population. On the last occasion of sampling, 8 lines were established. 4 of these belonged to the same clone that provided the earlier lines, 4 other lines grew from another clone that was infected *in vivo* with a different EBV sub-strain. The detection of EBV encoded proteins indicated that these cells were Type I or IIa cells. Because they seemed to lack EBNA-2. It seems therefore that the CLL cells that acquired the virus *in vivo* expressed the growth program *in vitro*, probably because they were released from the immunological control.

In the ex vivo sample that gave rise to the MEC lines, DNA encoding EBNA-2 was not detected.[20] Therefore the authors favored the possibility that infection of the CLL cells occurred *in vitro* by virus released from normal B cells in the culture. It cannot be ruled out however that the viral EBNA-2 code present in very few cells in the ex vivo sample evaded detection. Though we have no direct evidence for presence of the EBV infected cells in the CLL population, we like to consider this for discussion.

The analysis of the EBV terminal repeat and the EBNA-2 promoter expression in the lines indicated that the cells of origin were infected *in vivo* and at different occasions. The following scenario can be proposed. The CLL cell which was the origin of the line entered into a differentiation state that allowed the expression of the EBV encoded growth program but T cell derived factors, suppressed one or both proteins pivotal for proliferation (EBNA-2 and LMP-1). The cell then followed its own EBV independent proliferation dynamic *in vivo*. However, since CLL cells do not proliferate *in vitro*, upon explantation the rare EBV carrying cells were selected in the culture. This assumption may be justified by the cessation of proliferation and deregulation of the viral growth program when the cells were treated with IL-21 or with CD40L. *In vitro* experiments and the emergence of EBV positive proliferating B cell malignancies in immunosuppressive conditions indicate that the EBV carrying B cells can be controlled by immunological mechanisms [4].

The important massage of this work is that the viral gene harboring lines reflect the characteristics of their cell of origin. The phenotypic difference between the two MEC lines represents two considerably different phases of the CLL to PLL transition. Analysis of the MEC1 cell population showed already conspicuous change for some of the markers. For others, the population was still heterogeneous. MEC2 was established when the lymphocyte count was very high and the PLL character was evident.

The unusual phenotype of the MEC1 line deserves attention. While it expresses the growth program, Type III, CD23 is expressed by a smaller proportion of cells and it does not express the activation marker CD38. The MEC1 line provides an eminent example for the differential assortment of markers related to the EBV induced biological behavior. The properties of the two lines exemplify the complexity of EBV and the target interaction regulated by the differentiation of the B cell. Based on phenotypic marker expression, the MEC1 line would be a mixture of virus carrying cells in different phases of CLL progression towards PLL, while MEC2 would represent the fully developed PLL.

The two MEC lines were utilized in several studies for different objectives.[21], [22], [23], [59], [60] A noteworthy difference related to the characteristics of the disease conditions was found when the cells were inoculated to Rag2−/−γc−/− mice. MEC1 cells were detected in bone marrow, blood, lymph node and peritoneum.[22] The engrafted MEC2 cells did not grow.[23] In this respect the MEC lines conformed with CLL versus LCLs established from normal B cells. MEC1 has been stated to behave like CLL cells while MEC2 similar to LCLs do not establish as tumors in immunocompromised mice [61].

We emphasize 3 aspects of the characteristics of the MEC lines. 1. The EBV encoded growth program was expressed by both lines but the cells differed in phenotype and the MEC1 line retained some features of CLL cells. These are so prominent that the line was used in several studies as representative for CLL cells. Thus the expression of EBV in the MEC1 line did not override the B phenotype. 2. The expression of the incoming EBV gene can be determined by the event of infection. 3. The EBV carrying CLL cells do not express the Type III growth program *in vivo* even in a serious state of the disease indicating the immunological control is still in function.

## Supporting Information

Figure S1 **Terminal repeat analysis of MEC1 and MEC2 cell line.**
(TIF)

Figure S2 **Staining with secondary antibody and comparison of cell size by FSC.**
(TIF)

Figure S3 **Nucleic acid sequences of Cp after bisulfite-modification.** Overlapping raw sequencing data of bisulfite-modified DNAs of the MEC1 and MEC2 lines, from nucleotide 10664 to 11341, according to the prototype B95-8 sequence.[34] Boxes indicate the positions of the CBF1 and CBF2 binding sites. Green line: adenine; blue line: cytosine; black line: guanine; red line: thymine.
(PPTX)

Table S1 **Primers used in PCR.**
(DOC)

Table S2 **Phenotypic analysis of MEC1 and MEC2.**
(DOC)
