# Supplementary figures and images for: The MEC1 and MEC2 Lines Represent Two CLL Subclones in Different Stages of Progression towards Prolymphocytic Leukemia
Source: PLoS One. 2014 Aug 27;9(8):e106008. doi: 10.1371/journal.pone.0106008 (PMC4146575; doi:10.1371/journal.pone.0106008)

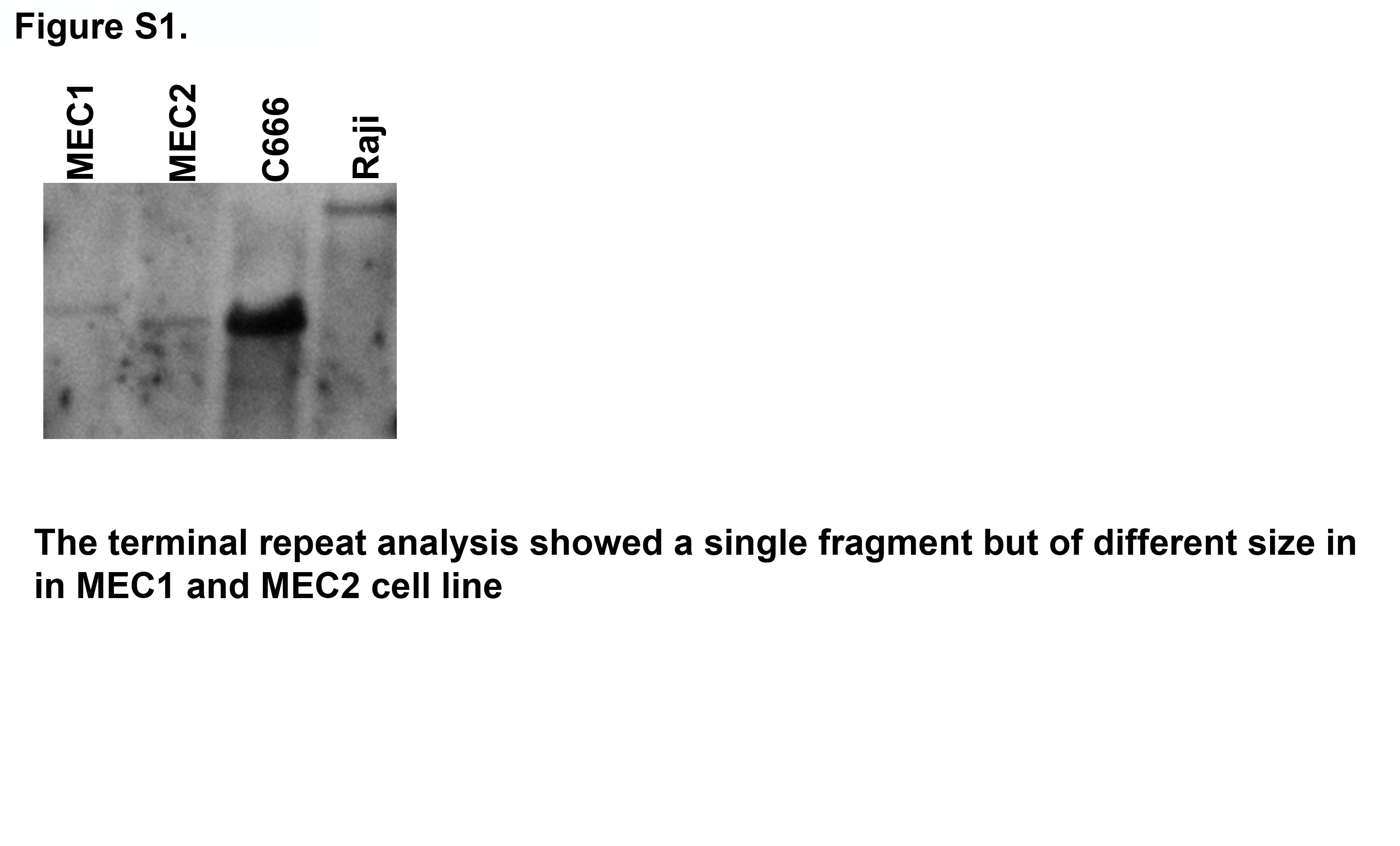

Supplement: Figure S1 — Terminal repeat analysis of MEC1 and MEC2 cell line. (TIF) [file pone.0106008.s001.tif]

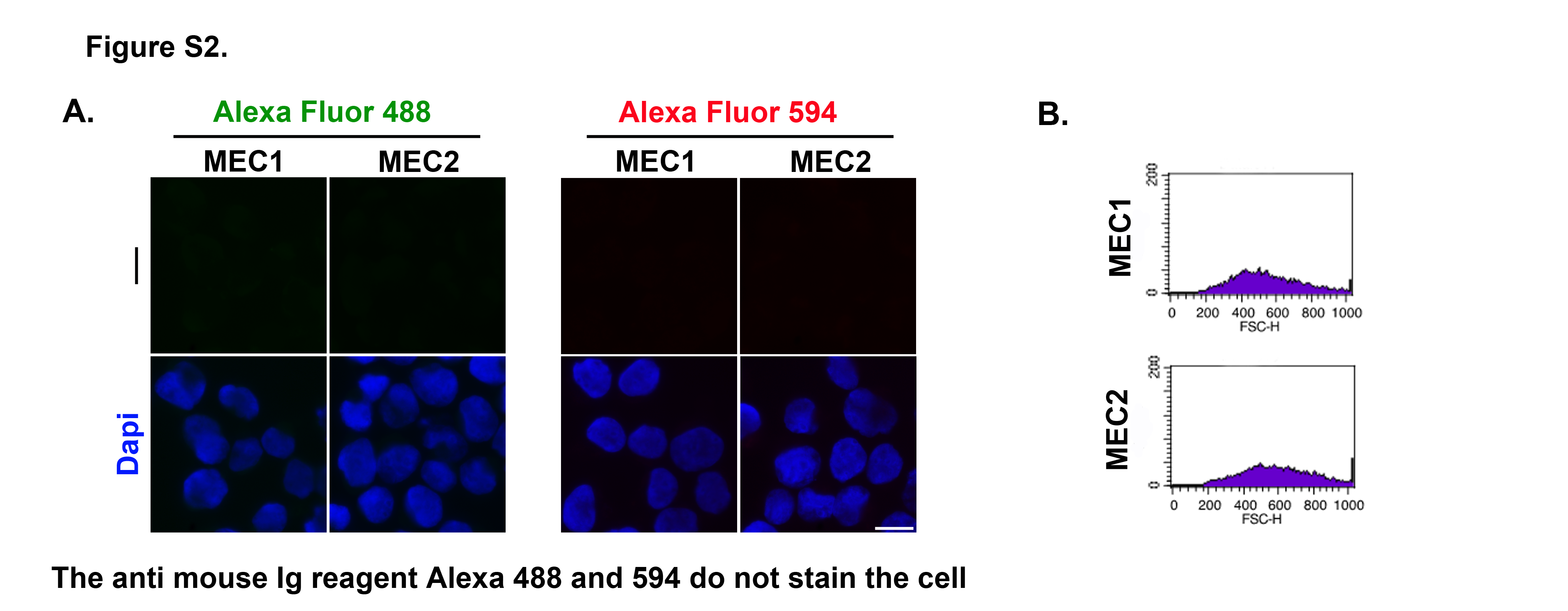

Supplement: Figure S2 — Staining with secondary antibody and comparison of cell size by FSC. (TIF) [file pone.0106008.s002.tif]

## Slide 1
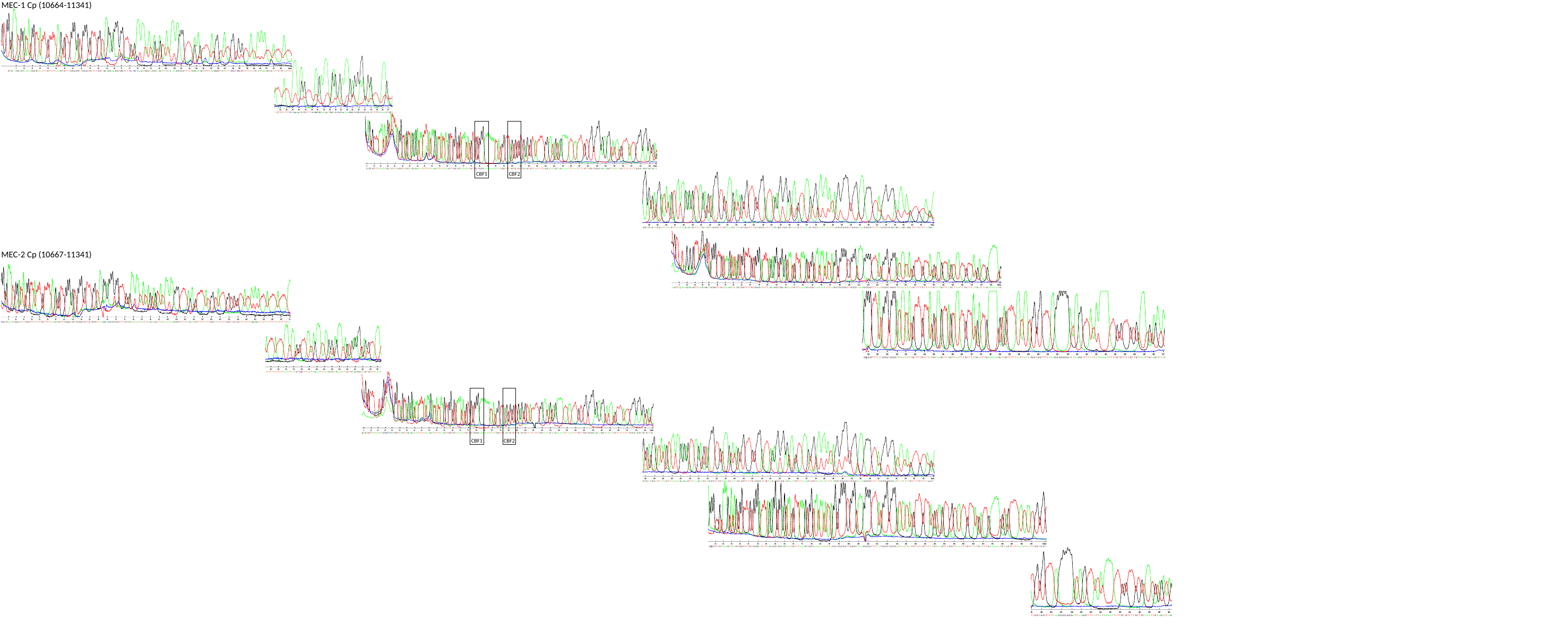

MEC-1 Cp (10664-11341)
CBF2
CBF1
MEC-2 Cp (10667-11341)
CBF2
CBF1

Supplement: Figure S3 — Nucleic acid sequences of Cp after bisulfite-modification. Overlapping raw sequencing data of bisulfite-modified DNAs of the MEC1 and MEC2 lines, from nucleotide 10664 to 11341, according to the prototype B95-8 sequence.[34] Boxes indicate the positions of the CBF1 and CBF2 binding sites. Green line: adenine; blue line: cytosine; black line: guanine; red line: thymine. (PPTX) [file pone.0106008.s003.pptx]
