# Supplementary material for: The MEC1 and MEC2 Lines Represent Two CLL Subclones in Different Stages of Progression towards Prolymphocytic Leukemia
Source: PLoS One. 2014 Aug 27;9(8):e106008. doi: 10.1371/journal.pone.0106008 (PMC4146575; doi:10.1371/journal.pone.0106008)
Supplement: Table S1 — Primers used in PCR. (DOC) [file pone.0106008.s004.doc]

**Table S1.**

Primers used in PCR

**Primers for real-time RT-PCR:**

Cp 5’-GATCAGATGGCATAGAGACAAGGAC-3’

5’AGGCTGTTTCTTCAGTCGGTTTAG--3’

Wp 5’-CGCCAGGAGTCCACACAAAT-3’

5’-GAGGGGACCCTCTGGCC-3’

Qp 5’-GATAGCGTGCGCTACCGGAT-3’

5’-TGCAGAATCAGCTCTCCCAAAC-3’

LMP1 5’-GCAGGAGGGTGATCATCAGT-3’

5’-GTCCTCTATTCCTTTGCTCTCATG-3’

GAPDH 5’-GGAAGGTGAAGGTCGGAGTCA-3’

5’-ATGGGTGGAATCATATTGGAACA-3’

**Primers for PCR amplification of unmodified Cp:**

5’-GCTTAACGGGAAGAGAAGTGG-3’

5’-CTTACGGTTTAGATGATTTGTGG-3’

**Primers for PCR amplification of bisulfite-modified Cp:**

outer 5’-GGGTTTAGGTTTTGTAGGGTAGA-3’

5’-CCCTACRATAAAAACTCTAAAAATCTT-3’

inner 5’-Univ-GTTAGGTTGATAAGGGGATAAG-3’

5’-Univ-TGAGAGGTTAGTGTTTTAAATATGT-3’

5’-Univ-GGATTATAGTTAATAAGAGAGTTTAAGA-3’

5’-Biotin-ATCCTTATCTCTATACCATCTAATCTA-3’

Primers were purchased from Metabion (Martinsried, Germany). Abbreviations: Univ, M13 universal primer sequence (5’-GTAAAACGACGGCCAGT-3’).
