# Supplementary material for: The MEC1 and MEC2 Lines Represent Two CLL Subclones in Different Stages of Progression towards Prolymphocytic Leukemia
Source: PLoS One. 2014 Aug 27;9(8):e106008. doi: 10.1371/journal.pone.0106008 (PMC4146575; doi:10.1371/journal.pone.0106008)
Supplement: Table S2 — Phenotypic analysis of MEC1 and MEC2. (DOC) [file pone.0106008.s005.doc]

**Table S2.**

Phenotypic analysis of MEC1 and MEC2

|  | MEC1 (%) | | MEC2 (%) | | Ex vivo Sample |
| --- | --- | --- | --- | --- | --- |
|  | Previous Findings[19] | Present Findings | Previous Findings[19] | Present Findings |  |
| HLA-ABC  (MHC class I) |  | 96 |  | 99 |  |
| HLA-DR (MHC class II) | 99 | 97,5 | 97 | 97,7 | 58 |
| IgA |  | <1 |  | <1 |  |
| CD5 | <1 | 0 |  | 0 | 4,5 |
| CD10 | <1 | 0 |  | 0 | <1 |
| CD19 | 99 | 99 | 96 | 99 | 66 |
| CD20 | 96 | 95 | 96 | 97 |  |
| CD21 | 58 | 91 | 59 | 56 |  |
| CD23 | 89 | 51 | 36 | 83 | 11.5 |
| CD25 | 9 | 0 | 3 | 0 |  |
| CD27 |  | 23 |  | 2 |  |
| CD30 | 83 | 78 | 30 | 81 | 11.5 |
| CD38 | 90 | <1 | 99 | 64 |  |
| IL-21R |  | 51 |  | 28 |  |
| CD11c | 13 | 0 | 11 | 0 | 27 |
| CD54/ICAM-1 | 98 | 90 | 98 | 95 | 3 |
| CD79a |  | 0 |  | 0 |  |
| CD95 | 90 | 99 | 63 | 99 |  |
| CXCR4 |  | 84 |  | 27 |  |
| CXCR5 |  | 0 |  | 0 |  |
| CCR7 |  | 99 |  | 92 |  |
| CCR10 |  | 75 |  | 26 |  |
| FMC7 | 8 | 74 | 72 | 97 | 81 |
